# Correlates of Regular Participation in Sports Groups among Japanese Older Adults: JAGES Cross–Sectional Study

**DOI:** 10.1371/journal.pone.0141638

**Published:** 2015-10-29

**Authors:** Mitsuya Yamakita, Satoru Kanamori, Naoki Kondo, Katsunori Kondo

**Affiliations:** 1 College of Liberal Arts and Sciences, Kitasato University, Kanagawa, Japan; 2 Department of Preventive Medicine and Public Health, Tokyo Medical University, Tokyo, Japan; 3 Human Resource Management Department, ITOCHU Techno–Solutions Corporation, Tokyo, Japan; 4 Departments of Health and Social Behavior/Health Education and Health Sociology, School of Public Health, University of Tokyo, Tokyo, Japan; 5 Center for Preventive Medical Science, Chiba University, Chiba, Japan; 6 Center for Well–being and Society, Nihon Fukushi University, Aichi, Japan; Chiba University Center for Forensic Mental Health, JAPAN

## Abstract

**Background:**

Participation in a sports group is key for the prevention of incident functional disability. Little is known about the correlates of older adults’ participation in sports groups, although this could assist with the development of effective health strategies. The purpose of this study was to identify the demographic and biological, psychosocial, behavioral, social and cultural, and environmental correlates of sports group participation among Japanese older adults.

**Methods:**

Data were obtained from the Japan Gerontological Evaluation study, which was a population–based cohort of people aged ≥65 years without disability enrolled from 31 municipalities across Japan (n = 78,002). Poisson regression analysis was used to determine the associations between the factors and participation in sports groups.

**Results:**

Non-regular participation in sports groups was associated with lower educational level, being employed, and working the longest in the agricultural/forestry/fishery industry among the demographic and biological factors and poor self-rated health and depression among the psychosocial factors. Of the behavioral factors, current smoking was negatively associated and current drinking was positively associated with regular participation in sports groups. Among the social and cultural factors, having emotional social support and participating in hobby clubs, senior citizen clubs, or volunteer groups were associated with a high prevalence of participation in sports groups. Perceptions of the presence of parks or sidewalks, good access to shops, and good accessibility to facilities were positively associated with participation in sports groups among the environmental factors.

**Conclusions:**

Our study suggests that the promotion of activities that could increase older adults’ participation in sports groups should consider a broad range of demographic and biological, psychosocial, behavioral, social and cultural, and environmental factors. Although future longitudinal studies to elucidate the causal associations are needed, encouraging participation in community groups through social networks might be effective for participation in sports groups.

## Introduction

The life expectancy of the global population has dramatically increased in recent years, and there is a corresponding growing public health concern regarding disability and the loss of physical and social function [1,2]. Although Japan has the longest life expectancy in the world [3], it also has the fastest increase in the aging population; 25.0% of the population is older than 65 years, and 17.6% of the older population has reported disabilities [4]. Therefore, reducing incident functional disability is an important goal for Japan and other countries with rapidly aging populations [5].

Studies have suggested that physical activity is an effective way to prevent incident functional disability [6]. Physical activity has many other potential health benefits, including prevention of various diseases [7]. Social participation is also suggested to prevent incident functional disability and has many health benefits for older adults, through social connectedness, social support, peer bonding, and self–esteem [8–10]. Based on these previous studies, “participation in sport groups” includes not only the physiological benefits of increased physical activity but also the psychosocial health benefits from social participation [9,11].

A study with older Japanese adults indicated that participation in sports groups was associated with a decreased likelihood of requiring long–term care when compared with individual exercise [12]. Participating in sports groups was also associated with the lowest risks for incident functional disability among eight types of social participation [13]. Moreover, participating in sports groups may reduce the risks for dementia [14], stroke [15], and falls [16] as well as functional decline. Therefore, promoting participation in sports groups might have many public health implications.

Because physical activity is a complex behavior determined by diverse factors, behavioral theories and models are used to guide the selection of variables to study [17]. A recent review classified the potential determinants into the following five categories [18], which have been associated with physical activity [17–20]: demographic and biological factors (e.g., age, sex, education, and occupation), psychosocial (e.g., self–efficacy), behavioral (e.g., previous activity participation), social and cultural (e.g., social support), and environmental factors (e.g., access to recreation facilities and locations, transport environment, and aesthetics). Because participation in sports groups includes the benefits of both physical activity and social participation, encouraging participation might be more likely to prevent functional disability than the promotion of physical activity. Thus, identifying the correlates of participation in sports group is essential to develop effective public health strategies. However, to the best of our knowledge, no studies have investigated the association between these potential determinants and participation in sports group among older adults.

Therefore, the purpose of the present study was to identify the demographic and biological, psychosocial, behavioral, social and cultural, and environmental correlates of participation in sports groups, using data from a large–scale cohort of Japanese older adults.

## Materials and Methods

### Study participants

Data from the Japan Gerontological Evaluation Study (JAGES) were utilized for this study. The JAGES project is an on–going prospective cohort study that aims to conduct empirical studies from gerontological and social epidemiological perspectives among individuals aged ≥65 years. The participants included those who did not already have a physical or cognitive disability, which was defined as not being eligible for public long–term care insurance benefits at baseline (i.e., registered disabled older people in municipality long–term care insurance systems were excluded).

From July 2010 to January 2012, a self–administered questionnaire was mailed to a random sample of 169,215 community–dwelling individuals aged ≥65 years residing in 31 municipalities in 12 prefectures in Japan. Of the eligible participants, 112,123 people returned the questionnaire (response rate, 66.3%). Participants with missing values for age or sex (n = 8,502) or participation in sports groups (n = 22,238) or who needed assistance in activities of daily living (n = 3,381) were excluded from the analysis, resulting in 78,002 participants. Missing responses to any of the other variables were categorized as “missing” for analyses. The JAGES protocol was reviewed and approved by the Ethics Committee on Research of Human Subjects at Nihon Fukushi University (Approval No. 10–05). Written informed consent was assumed with voluntary return of the questionnaire.

### Measures

#### Participation in sports groups

Participation in sport groups was assessed using the following question: “How often do you participate in a sports group or club?” Those who answered “almost every day,” “2 or 3 times a week,” or “once a week” were considered “regular participants,” and those who answered “once or twice a month,” “a few times a year,” or “never” were considered “non–regular participants.”

#### Selection and categorization of variables

Based on previous reviews of physical activity determinants [17–20], the measures on the questionnaire were selected and classified into the following five categories: demographic and biological, psychosocial, behavioral, social and cultural, or environmental factors.

#### Demographic and biological factors

The following data for demographic and biological factors were collected: sex, age, body mass index (BMI), marital status, educational level, household income, occupational status, the longest job, and instrumental activities of daily living (IADL). BMI was calculated from self–reported height and weight (kg/m^2^). IADL was assessed using the Tokyo Metropolitan Institute of Gerontology Index of Competence [21], and the results were classified as good (5 points) or poor (≤4 points). Household income, which was divided by the square root of household size and equivalized, was categorized into 3 groups (1.5 million yen = 12,500 US dollars): <1.5 million yen, 1.5 to <2.5 million yen, or ≥2.5 million yen. The longest job was categorized as clerical, administrative, professional/technical, sales/service, skilled/labor, agriculture/forestry/fishery, other, or no occupation [22].

#### Psychosocial factors

The following psychosocial factors were collected: self–rated health, depression, general trust, norms of reciprocity, and attachment to the neighborhood. Depression was measured using the short version of the Geriatric Depression Scale–15 [23] and was categorized into 3 groups: no (0–4 points), mild (5–9 points), or moderate to severe (10–15 points). General trust, norms of reciprocity, and attachment to the neighborhood were categorized as yes (very, moderately) or no (neutral, slightly, not at all).

#### Behavioral factors

Smoking status and alcohol intake were assessed as behavioral factors and categorized into 3 groups (never, ever, or current).

#### Social and cultural factors

Emotional and instrumental social support (both received and given), meeting friends, number of met friends, interactions with neighbors, and social participation were collected as social and cultural factors. Emotional and instrumental social support, both received and given, were dichotomized as yes or no. Frequency of meeting friends was categorized as almost every day, 2 or 3 times per week, once a week, once or twice per month, or a few times a year or less. Interactions with neighbors were categorized into 3 groups: cooperating in daily life, standing and chatting frequently, or no more than an exchange of greetings/none.

For social participation, respondents were asked whether they belonged to a political organization or group, industrial or trade association, volunteer group, senior citizen club, religious organization or group, neighborhood association or residents’ association, or hobby club (yes or no). These questions have been described in detail previously [13, 24].

#### Environmental factors

Environmental factors were evaluated using population density and perceived neighborhood environment. Population density was used as a regional characteristic and calculated on inhabitable land for each municipality and classified into 4 groups: metropolitan (≥4,000 people/km^2^), urban (1,500–3,999 people/km^2^), semi–urban (1,000–1,499 people/km^2^), or rural (<1000 people/km^2^) [24]. The perceived neighborhood environment was ascertained by asking whether any of the following 8 items were located within 1 km from their home: graffiti or garbage, parks or sidewalks, hills or steps, risk of traffic accidents, fascinating views or buildings, access to shops (shops or facilities selling fresh fruits and vegetables), risk of crime at night, or access to facilities (houses or facilities you feel free to drop in). All items were categorized as yes (“many” or “some”) or no (“few” or “none”).

### Statistical analysis

Following recommendations for the statistical analysis of binary outcomes [25], Poisson regression analyses with robust variance estimators were conducted to examine the associations between potential correlates and the participation in sports groups, which resulted in a prevalence ratio (PR) for each variable. All multivariable analyses were adjusted for socio–demographic and health–related covariates (Model 1: age, sex, BMI, marital status, educational level, household income, occupational status, IADL, self–rated health, depression, smoking status, and alcohol intake). In addition, population density variation was added to the model (Model 2) because, in theory, social and cultural as well as environmental factors might be strongly influenced by population density, which reflects the level of urbanization.

All statistical analyses were performed using SPSS version 19.0 (SPSS Inc., Chicago, IL, USA). A P–value of less than 0.05 (two–tailed) was considered statistically significant.

## Results

The mean age (standard deviation) of the regular participants was 73.5 (6.1) years; 48.4% were women, and 18.3% were considered regular participants (Table 1). In the crude and adjusted analyses, although the strengths and directions of the associations varied, as discussed in the following sections, all factors were significantly associated with participation in sports groups.

**Table 1:** Characteristics of the elderly respondents to the questionnaire regarding participation in sports groups.

	n	(%)
**Overall**	78,002	(100.0)
**Regular participation in sports groups**		
Regular Participants	14,302	(18.3)
Non–regular participants	63,700	(71.7)
***Demographic and biological factors***		
**Sex**		
Male	37,772	(48.4)
Female	40,230	(51.6)
**Age group (years)**		
65–69	24,429	(31.3)
70–74	23,289	(29.9)
75–79	16,565	(21.2)
80–84	9,282	(11.9)
≥85	4,437	(5.7)
**BMI (kg/m** ^**2**^ **)**		
18.5–24.9	53,391	(68.4)
<18.5	5,123	(6.6)
≥25.0	16,594	(21.3)
Missing	2,894	(3.7)
**Marital status**		
Married	56,454	(72.4)
Widowed	16,116	(20.7)
Divorced	2,474	(3.2)
Never married	1,578	(2.0)
Others and missing	1,380	(1.8)
**Educational level (years)**		
≥13	14,395	(18.5)
10–12	26,611	(34.1)
6–9	31,752	(40.7)
<6	1,784	(2.3)
Others and missing	3,460	(4.4)
**Equivalent household income (yen)**		
≥2.5 million	21,846	(28.0)
1.5–2.5 million	26,342	(33.8)
<1.5 million	17,889	(22.9)
Missing	11,925	(15.3)
**Occupational status**		
Employed	16,430	(21.1)
Retired/Not employed	44,287	(56.8)
Never employed	9,109	(11.7)
Missing	8,176	(10.5)
**Longest job**		
Clerical	11,289	(14.5)
Administrative	4,832	(6.2)
Professional/technical	12,004	(15.4)
Sales/service	10,618	(13.6)
Skilled/labor	10,073	(12.9)
Agriculture/forestry/fishery	5,731	(7.3)
Others	9,171	(11.8)
No occupation	4,158	(5.3)
Missing	10,126	(13.0)
**IADL**		
Good	58,783	(75.4)
Poor	14,146	(18.1)
Missing	5,073	(6.5)
***Psychosocial factors***		
**Self–rated health**		
Very good	9,281	(11.9)
Good	53,367	(68.4)
Poor	12,611	(16.2)
Very poor	1,941	(2.5)
Missing	802	(1.0)
**Depression**		
No	47,876	(61.4)
Mild	13,521	(17.3)
Mild to severe	4,409	(5.7)
Missing	12,196	(15.6)
**General trust**		
No	20,871	(26.8)
Yes	54,431	(69.8)
Missing	2,700	(3.5)
**Norms of reciprocity**		
No	32,625	(41.8)
Yes	43,711	(56.0)
Missing	1,666	(2.1)
**Attachment to the neighborhood**		
No	13,789	(17.7)
Yes	63,028	(80.8)
Missing	1,185	(1.5)
***Behavioral factors***		
**Smoking status**		
Never	42,371	(54.3)
Ever	21,050	(27.0)
Current	8,036	(10.3)
Missing	6,545	(8.4)
**Alcohol intake**		
Never	44,295	(56.8)
Ever	2,561	(3.3)
Current	27,184	(34.9)
Missing	3,962	(5.1)
***Social and cultural factors***		
**Receiving emotional support**		
No	4,589	(5.9)
Yes	71,963	(92.3)
Missing	1,450	(1.9)
**Providing emotional support**		
No	5,641	(7.2)
Yes	70,552	(90.4)
Missing	1,809	(2.3)
**Receiving instrumental support**		
No	3,783	(4.8)
Yes	73,004	(93.6)
Missing	1,215	(1.6)
**Providing instrumental support**		
No	9,115	(11.7)
Yes	66,392	(85.1)
Missing	2,495	(3.2)
**Frequency of meeting friends**		
A few times a year or less	19,659	(25.2)
1–2 times/month	15,328	(19.7)
About once/week	12,873	(16.5)
2–3 times/week	17,362	(22.3)
Almost every day	10,622	(13.6)
Missing	2,158	(2.8)
**Number of met friends**		
0	4,634	(5.9)
1–2	12,440	(15.9)
3–5	18,826	(24.1)
6–9	10,542	(13.5)
≥10	28,744	(36.9)
Missing	2,816	(3.6)
**Interactions with neighbors**		
Cooperating in daily life	12,766	(16.4)
Standing and chatting frequently	42,250	(54.2)
No more than an exchange of greetings/none	17,871	(22.9)
Missing	5,115	(6.6)
**Social participation**		
**Politics**		
No	53,904	(69.1)
Yes	7,933	(10.2)
Missing	16,165	(20.7)
**Industry**		
No	58,823	(75.4)
Yes	11,544	(14.8)
Missing	7,635	(9.8)
**Volunteer**		
No	58,624	(75.2)
Yes	13,295	(17.0)
Missing	6,083	(7.8)
**Senior citizen club**		
No	55,796	(71.5)
Yes	18,594	(23.8)
Missing	3,612	(4.6)
**Religion**		
No	54,031	(69.3)
Yes	9,281	(11.9)
Missing	14,690	(18.8)
**Neighborhood community**		
No	43,859	(56.2)
Yes	30,932	(39.7)
Missing	3,211	(4.1)
**Hobby**		
No	42,444	(54.4)
Yes	32,998	(42.3)
Missing	2,560	(3.3)
***Environmental factors***		
**Population density**		
Metropolitan	16,720	(21.4)
Urban	16,856	(21.6)
Semi–urban	17,227	(22.1)
Rural	27,199	(34.9)
**Perceived neighborhood environment**		
**Graffiti or garbage**		
No	49,955	(64.0)
Yes	20,998	(26.9)
Missing	7,049	(9.0)
**Parks or sidewalks**		
No	20,708	(26.5)
Yes	53,765	(68.9)
Missing	3,529	(4.5)
**Hills or steps**		
No	43,905	(56.3)
Yes	30,864	(39.6)
Missing	3,233	(4.1)
**Risk of traffic accidents**		
No	24,126	(30.9)
Yes	50,365	(64.6)
Missing	3,511	(4.5)
**Fascinating views or buildings**		
No	42,043	(53.9)
Yes	30,455	(39.0)
Missing	5,504	(7.1)
**Access to shops**		
No	19,248	(24.7)
Yes	56,412	(72.3)
Missing	2,342	(3.0)
**Risk of crime at night**		
No	24,622	(31.6)
Yes	46,187	(59.2)
Missing	7,193	(9.2)
**Access to facilities**		
No	40,526	(52.0)
Yes	30,077	(38.6)
Missing	7,399	(9.5)

IADL, instrumental activities of daily living; BMI, body mass index

### Demographic and biological factors

Table 2 shows the PRs for participation in sports groups according to demographic and biological factors. In the adjusted Model 1, male sex, older age, underweight (BMI <18.5 kg/m^2^) and obesity (BMI ≥25 kg/m^2^), never being married, lower educational level, lower household income, employment, and low IADL score were negatively associated with participation in sports groups. Among the jobs held the longest, the PR of agriculture/forestry/fishery work was significantly lower than that of clerical work (PR, 0.55; 95% confidence interval [CI], 0.50–0.59).

**Table 2:** Associations between participating in sports groups and demographic and biological factors.

	Regular participation in sports groups	Crude analysis	Adjusted analysis (Model 1^a^)
	n (%)	PR (95% CI)	PR (95% CI)
**Sex**			
Male	6,075 (16.1)	ref	ref
Female	8,227 (20.4)	1.27 (1.23–1.31)	1.40 (1.35–1.45)
**Age (years)**			
65–69	4,830 (19.8)	ref	ref
70–74	4,740 (20.4)	1.03 (0.99–1.07)	1.08 (1.04–1.12)
75–79	3,126 (18.9)	0.95 (0.92–0.99)	1.07 (1.02–1.11)
80–84	1,200 (12.9)	0.65 (0.62–0.69)	0.78 (0.73–0.83)
≥85	406 (9.2)	0.46 (0.42–0.51)	0.59 (0.54–0.65)
**BMI (kg/m** ^**2**^ **)**			
18.5–24.9	10,413 (19.5)	ref	ref
<18.5	691 (13.5)	0.69 (0.64–0.74)	0.75 (0.70–0.81)
≥25.0	2,859 (17.2)	0.88 (0.85–0.92)	0.92 (0.88–0.95)
Missing	339 (11.7)	0.60 (0.54–0.66)	0.75 (0.67–0.83)
**Marital status**			
Married	10,794 (19.1)	ref	ref
Widowed	2,820 (17.5)	0.92 (0.88–0.95)	0.94 (0.91–0.98)
Divorced	315 (12.7)	0.67 (0.60–0.74)	0.69 (0.62–0.76)
Never married	204 (12.9)	0.68 (0.59–0.77)	0.69 (0.61–0.78)
Others and missing	169 (12.2)	0.84 (0.78–0.91)	0.79 (0.68–0.91)
**Educational level (years)**			
≥13	3,191 (22.2)	ref	ref
10–12	5,572 (20.9)	0.94 (0.91–0.98)	0.94 (0.90–0.97)
6–9	4,762 (15.0)	0.68 (0.65–0.70)	0.75 (0.72–0.78)
<6	131 (7.3)	0.33 (0.28–0.39)	0.44 (0.37–0.52)
Others and missing	646 (18.7)	0.84 (0.78–0.91)	0.93 (0.87–1.01)
**Equivalent household income (yen)**			
≥2.5 million	4,728 (21.6)	ref	ref
1.5–2.5 million	5,308 (20.2)	0.93 (0.90–0.96)	0.96 (0.92–0.90)
<1.5 million	2,378 (13.3)	0.61 (0.59–0.64)	0.69 (0.66–0.72)
Missing	1,888 (15.8)	0.73 (0.70–0.77)	0.83 (0.79–0.87)
**Occupational status**			
Employed	2,214 (13.5)	ref	ref
Retired/not employed	9,181 (20.7)	1.54 (1.47–1.61)	1.70 (1.62–1.77)
Never employed	1,526 (16.8)	1.24 (1.17–1.32)	1.42 (1.34–1.52)
Missing	1,381 (16.9)	1.25 (1.18–1.33)	1.61 (1.51–1.72)
**Longest job**			
Clerical	2,828 (25.1)	ref	ref
Administrative	1,072 (22.2)	0.89 (0.83–0.94)	1.03 (0.97–1.10)
Professional/technical	2,373 (19.8)	0.79 (0.75–0.83)	0.91 (0.87–0.96)
Sales/service	1,875 (17.7)	0.70 (067–0.74)	0.79 (0.75–0.84)
Skilled/labor	1,718 (17.1)	0.68 (0.65–0.72)	0.88 (0.83–0.93)
Agriculture/forestry/fishery	606 (10.6)	0.42 (0.39–0.46)	0.55 (0.50–0.59)
Others	1,318 (14.4)	0.57 (0.54–0.61)	0.68 (0.64–0.73)
No occupation	720 (17.3)	0.69 (0.64–0.74)	0.67 (0.62–0.72)
Missing	1,792 (17.7)	0.71 (0.67–0.74)	0.74 (0.70–0.79)
**IADL**			
Good	12,063 (20.5)	ref	ref
Poor	1,449 (10.2)	0.50 (0.47–0.53)	0.62 (0.59–0.66)
Missing	790 (15.6)	0.76 (0.71–0.81)	1.01 (0.94–1.08)

PR, prevalence ratio; CI, confidence interval; ref, reference; BMI, body mass index; IADL, instrumental activities of daily living

^a^Model 1 is adjusted for age, sex, BMI, marital status, educational level, household income, occupational status, IADL, self–rated health, depression, smoking status, and alcohol intake.

### Psychosocial factors

Very poor self–rated health and mild to severe depression were associated with lower participation in sports groups in the adjusted analysis (Table 3). The adjusted PRs were 0.31 (95% CI, 0.26–0.37) and 0.43 (95% CI, 0.39–0.48), respectively. General trust, norms of reciprocity, and attachment to the neighborhood were positively associated with participating in sports groups.

**Table 3:** Associations between participating in sports groups and psychosocial factors.

	Regular participation in sports groups	Crude analysis	Adjusted analysis (Model 1^a^)
	n (%)	PR (95% CI)	PR (95% CI)
**Self–rated health**			
Very good	2,513 (27.1)	ref	ref
Good	10,241 (19.2)	0.71 (0.68–0.74)	0.74 (0.72–0.77)
Poor	1,307 (10.4)	0.38 (0.36–0.41)	0.45 (0.43–0.48)
Very poor	127 (6.5)	0.24 (0.20–0.29)	0.31 (0.26–0.37)
Missing	114 (14.2)	0.52 (0.44–0.62)	0.60 (0.50–0.71)
**Depression**			
No	10,146 (21.2)	ref	ref
Mild	1,638 (12.1)	0.57 (0.54–0.60)	0.68 (0.65–0.71)
Mild to severe	295 (6.7)	0.32 (0.28–0.35)	0.43 (0.39–0.48)
Missing	2,223 (18.2)	0.86 (0.83–0.90)	0.95 (0.92–1.00)
**General trust**			
No	3,003 (14.4)	ref	ref
Yes	10,790 (19.8)	1.38 (1.33–1.43)	1.26 (1.21–1.31)
Missing	509 (18.9)	1.31 (1.20–1.43)	1.40 (1.29–1.53)
**Norms of reciprocity**			
No	5,280 (16.2)	ref	ref
Yes	8,757 (20.0)	1.24 (1.20–1.28)	1.16 (1.13–1.20)
Missing	265 (15.9)	0.98 (0.88–1.10)	1.11 (0.99–1.24)
**Attachment to the neighborhood**			
No	1,759 (12.8)	ref	ref
Yes	12,353 (19.6)	1.54 (1.47–1.61)	1.40 (1.34–1.47)
Missing	190 (16.0)	1.26 (1.10–1.44)	1.40 (1.22–1.60)

PR, prevalence ratio; CI, confidence interval; ref, reference

^a^Model 1 is adjusted for age, sex, BMI, marital status, educational level, household income, occupational status, IADL, self–rated health, depression, smoking status, and alcohol intake.

### Behavioral factors

In the adjusted analysis, current smoking was negatively associated with participation in sports groups (PR, 0.68; 95% CI, 0.64–0.73; Table 4), while current drinking was positively associated with participation in sports groups (PR, 1.32; 95% CI, 1.27–1.36).

**Table 4:** Associations between participating in sports groups and behavioral factors.

	Regular participation in sports groups	Crude analysis	Adjusted analysis (Model 1^a^)
	n (%)	PR (95% CI)	PR (95% CI)
**Smoking status**			
Never	8478 (20.0)	ref	ref
Ever	3646 (17.3)	0.87 (0.84–0.90)	0.97 (0.92–1.01)
Current	986 (12.3)	0.61 (0.58–0.65)	0.68 (0.64–0.73)
Missing	1192 (18.2)	0.91 (0.86–0.96)	1.32 (1.25–1.40)
**Alcohol intake**			
Never	7557 (17.1)	ref	ref
Ever	343 (13.4)	0.79 (0.71–0.87)	1.06 (0.96–1.18)
Current	5618 (20.7)	1.21 (1.17–1.25)	1.32 (1.27–1.36)
Missing	784 (19.8)	1.16 (1.09–1.24)	2.03 (1.87–2.19)

PR, prevalence ratio; CI, confidence interval; ref, reference

^a^Model 1 is adjusted for age, sex, BMI, marital status, educational level, household income, occupational status, IADL, self–rated health, depression, smoking status, and alcohol intake.

### Social and cultural factors

All social and cultural factors were associated with participation in sports groups in the crude and both adjusted (Models 1 and 2) analyses (Table 5). In particular, emotional support (both receiving and providing), meeting friends, and number of met friends were significantly associated with participating in sports groups. The adjusted PRs in Model 2 were 1.64 (95% CI, 1.50–1.80) and 1.79 (95% CI, 1.64–1.96) for receiving and providing emotional support, respectively. In Model 2, all social participation factors were associated with participation in sports groups. In particular, participants in hobby clubs were 5.04 times (95% CI, 4.83–5.27) more likely to participate in sports groups than people who did not participate in hobby clubs. The next highest PRs were observed for participants in senior citizen clubs (PR, 2.51; 95% CI, 2.43–2.59), followed by participants in volunteer groups (PR, 1.96; 95% CI, 1.89–2.02).

**Table 5:** Associations between participating in sports groups and social and cultural factors.

	Regular participation in sports groups	Crude analysis	Model 1^a^	Model 2^b^
	n (%)	PR (95% CI)	PR (95% CI)	PR (95% CI)
**Receiving emotional support**				
No	422 (9.2)	ref	ref	ref
Yes	13,658 (19.0)	2.06 (1.88–2.26)	1.62 (1.48–1.78)	1.64 (1.50–1.80)
Missing	222 (15.3)	1.66 (1.43–1.94)	1.59 (1.37–1.85)	1.65 (1.42–1.91)
**Providing emotional support**				
No	466 (8.3)	ref	ref	ref
Yes	13,568 (19.2)	2.33 (2.13–2.54)	1.78 (1.63–1.95)	1.79 (1.64–1.96)
Missing	268 (14.8)	1.79 (1.56–2.06)	1.70 (1.48–1.95)	1.76 (1.53–2.02)
**Receiving instrumental support**				
No	505 (13.3)	ref	ref	ref
Yes	13,599 (18.6)	1.40 (1.28–1.52)	1.14 (1.05–1.24)	1.17 (1.08–1.27)
Missing	198 (16.3)	1.22 (1.05–1.42)	1.15 (0.99–1.34)	1.21 (1.04–1.40)
**Providing instrumental support**				
No	990 (10.9)	ref	ref	ref
Yes	12,917 (19.5)	1.79 (1.69–1.90)	1.43 (1.34–1.52)	1.44 (1.35–1.54)
Missing	395 (15.8)	1.46 (1.31–1.62)	1.46 (1.31–1.63)	1.50 (1.35–1.67)
**Frequency of meeting friends**				
A few times a year or less	1,112 (5.7)	ref	ref	ref
1–2 times/month	1,724 (11.2)	1.99 (1.85–2.14)	1.82 (1.70–1.96)	1.85 (1.72–1.99)
About once/week	2,652 (20.6)	3.64 (3.41–3.89)	3.28 (3.07–3.51)	3.32 (3.10–3.54)
2–3 times/week	5,656 (32.6)	5.76 (5.42–6.12)	5.15 (4.84–5.48)	5.23 (4.92–5.56)
Almost every day	2,782 (26.2)	4.63 (4.34–4.94)	4.17 (3.90–4.45)	4.24 (3.97–4.52)
Missing	376 (17.4)	3.08 (2.76–3.43)	3.27 (2.94–3.64)	3.39 (3.04–3.77)
**Number of met friends**				
0	172 (3.7)	ref	ref	ref
1–2	909 (7.3)	1.97 (1.68–2.31)	1.85 (1.58–2.17)	1.90 (1.62–2.23)
3–5	2,514 (13.4)	3.60 (3.09–4.18)	3.16 (2.72–3.68)	3.29 (2.83–3.82)
6–9	1,946 (18.5)	4.97 (4.27–5.79)	4.18 (3.59–4.87)	4.35 (3.73–5.06)
≥10	8,427 (29.3)	7.90 (6.81–9.16)	6.35 (5.47–7.36)	6.61 (5.70–7.67)
Missing	334 (11.9)	3.20 (2.67–3.82)	3.17 (2.65–3.78)	3.35 (2.81–4.00)
**Interactions with neighbors**				
Cooperating in daily life	2,667 (20.9)	ref	ref	ref
Standing and chatting frequently	8,185 (19.4)	0.93 (0.89–0.96)	0.94 (0.90–0.98)	0.89 (0.86–0.93)
No more than exchange greetings/none	2,451 (13.7)	0.66 (0.62–0.69)	0.74 (0.70–0.77)	0.67 (0.63–0.70)
Missing	999 (19.5)	0.93 (0.88–1.00)	1.03 (0.97–1.10)	0.98 (0.91–1.04)
**Social participation**				
**Politics**				
No	8,084 (15.0)	ref	ref	ref
Yes	1,974 (24.9)	1.66 (1.59–1.73)	1.64 (1.57–1.71)	1.65 (1.58–1.72)
Missing	4,244 (26.3)	1.75 (1.69–1.81)	1.84 (1.78–1.90)	1.78 (1.72–1.85)
**Industry**				
No	8,774 (14.9)	ref	ref	ref
Yes	2,570 (22.3)	1.49 (1.44–1.55)	1.43 (1.37–1.49)	1.45 (1.39–1.51)
Missing	2,958 (38.7)	2.60 (2.51–2.69)	2.78 (2.69–2.88)	2.84 (2.75–2.94)
**Volunteer**				
No	7,874 (13.4)	ref	ref	ref
Yes	3,920 (29.5)	2.20 (2.12–2.27)	1.91 (1.84–1.97)	1.96 (1.89–2.02)
Missing	2,508 (41.2)	3.07 (2.96–3.18)	3.31 (3.19–3.43)	3.36 (3.25–3.49)
**Senior citizen club**				
No	7,360 (13.2)	ref	ref	ref
Yes	5,326 (28.6)	2.17 (2.10–2.24)	2.35 (2.28–2.42)	2.51 (2.43–2.59)
Missing	1,616 (44.7)	3.39 (3.25–3.54)	3.64 (3.49–3.79)	3.65 (3.50–3.80)
**Religion**				
No	8,531 (15.8)	ref	ref	ref
Yes	1,667 (18.0)	1.14 (1.08–1.19)	1.15 (1.09–1.20)	1.15 (1.09–1.20)
Missing	4,104 (27.9)	1.77 (1.71–1.83)	1.85 (1.80–1.91)	1.87 (1.80–1.94)
**Neighborhood community**				
No	5,628 (12.8)	ref	ref	ref
Yes	6,826 (22.1)	1.72 (1.67–1.78)	1.57 (1.52–1.62)	1.65 (1.60–1.70)
Missing	1,848 (57.6)	4.49 (4.32–4.66)	4.39 (4.22–4.57)	4.49 (4.32–4.67)
**Hobby**				
No	2,422 (5.7)	ref	ref	ref
Yes	10,736 (32.5)	5.70 (5.47–5.94)	5.07 (4.86–5.30)	5.04 (4.83–5.27)
Missing	1,144 (44.7)	7.83 (7.39–8.30)	7.65 (7.23–8.11)	7.75 (7.32–8.21)

PR, prevalence ratio; CI, confidence interval; ref, reference

^a^Model 1 is adjusted for age, sex, BMI, marital status, educational level, household income, occupational status, IADL, self–rated health, depression, smoking status, and alcohol intake.

^b^Model 2 is adjusted for the factors in Model 1 plus population density.

### Environmental factors

Low population density (semi–urban, rural) was associated with lower participation in sports groups (Table 6). All of the perceived neighborhood environmental factors except hills or steps were positively associated with participation in sports groups in Model 1. Although further adjustment for population density attenuated these associations slightly, the significant associations persisted in Model 2. In particular, access to facilities was associated with the largest effect for participation in sports groups (PR, 1.25; 95% CI, 1.21–1.29), followed by access to shops (PR, 1.18; 95% CI, 1.13–1.22) and parks and sidewalks (PR, 1.14; 95% CI, 1.10–1.18).

**Table 6:** Associations between participating in sports groups and environmental factors in elderly respondents.

	Regular participation in sports groups	Crude analysis	Model 1^a^	Model 2^b^
	n (%)	PR (95% CI)	PR (95% CI)	PR (95% CI)
**Population density**				
Metropolitan	3,452 (20.6)	ref	ref	
Urban	3,738 (22.2)	1.07 (1.03–1.12)	1.06 (1.02–1.10)	
Semi––urban	3,036 (17.6)	0.85 (0.82––0.89)	0.88 (0.84–0.92)	
Rural	4,076 (15.0)	0.73 (0.70–0.76)	0.77 (0.74–0.80)	
**Perceived neighborhood environment**				
**Graffiti or garbage**				
No	9,391 (18.8)	ref	ref	ref
Yes	4,048 (19.3)	1.03 (0.99–1.06)	1.06 (1.02–1.09)	1.05 (1.02–1.09)
Missing	863 (12.2)	0.65 (0.61–0.70)	0.78 (0.74–0.84)	0.78 (0.73–0.83)
**Parks or sidewalks**				
No	3,273 (15.8)	ref	ref	ref
Yes	10,680 (19.9)	1.26 (1.21–1.30)	1.18 (1.14–1.23)	1.14 (1.10–1.18)
Missing	349 (9.9)	0.63 (0.56–0.69)	0.75 (0.68–0.83)	0.74 (0.67–0.82)
**Hills or steps**				
No	8,317 (18.9)	ref	ref	ref
Yes	5,595 (18.1)	0.96 (0.93–0.99)	1.00 (0.97–1.03)	0.98 (0.95–1.01)
Missing	390 (12.1)	0.64 (0.58–0.70)	0.79 (0.72–0.87)	0.80 (0.73–0.88)
**Risk of traffic accidents**				
No	4,201 (17.4)	ref	ref	ref
Yes	9,654 (19.2)	1.10 (1.07–1.14)	1.12 (1.08–1.16)	1.11 (1.07–1.14)
Missing	447 (12.7)	0.73 (0.67–0.80)	0.89 (0.82–0.98)	0.90 (0.82–0.98)
**Fascinating views or buildings**				
No	7,347 (18.5)	ref	ref	ref
Yes	6,285 (20.6)	1.18 (1.15–1.22)	1.11 (1.08–1.15)	1.11 (1.07–1.14)
Missing	670 (12.2)	0.70 (0.65–0.75)	0.82 (0.76–0.89)	0.83 (0.77–0.89)
**Access to shops**				
No	2,926 (15.2)	ref	ref	ref
Yes	11,086 (19.7)	1.29 (1.25–1.34)	1.21 (1.17–1.26)	1.18 (1.13–1.22)
Missing	290 (12.4)	0.81 (0.73–0.91)	0.98 (0.87–1.09)	0.97 (0.87–1.08)
**Risk of crime at night**				
No	4,424 (18.0)	ref	ref	ref
Yes	8,948 (19.4)	1.08 (1.04–1.11)	1.07 (1.03–1.10)	1.05 (1.01–1.08)
Missing	930 (12.9)	0.72 (0.67–0.77)	0.82 (0.77–0.87)	0.82 (0.77–0.87)
**Access to facilities**				
No	6,845 (16.9)	ref	ref	ref
Yes	6,524 (21.7)	1.28 (1.25–1.32)	1.23 (1.19–1.26)	1.25 (1.21–1.29)
Missing	933 (12.6)	0.75 (0.70–0.80)	0.83 (0.78–0.88)	0.82 (0.77–0.87)

PR, prevalence ratio; CI, confidence interval; ref, reference

^a^Model 1 is adjusted for age, sex, BMI, marital status, educational level, household income, occupational status, IADL, self–rated health, depression, smoking status, and alcohol intake.

^b^Model 2 is adjusted for the factors in Model 1 plus population density.

## Discussion

All of the examined factors in the present study were associated with participation in sports groups in Japanese older adults. The demographic and biological factors (i.e., education, occupation, and IADL), psychosocial (i.e., self–rated health and depression), and social and cultural factors (i.e., social support and social participation) showed particularly strong associations with participation in sports groups.

Although these trends were mostly similar to the results of recent studies on the correlates of physical activity [17–20], we did find some differences in the associations with participation in sports groups. First, men were less likely to participate in sports groups than women; this finding is different from that of other studies in which men were more likely to participate in physical activity [17–20, 26]. However, other studies have also reported that men are less likely to participate in community–based physical activity programs [27] and services that emphasized social interaction [28]; these findings indicate that there might be gender differences in social participation. Collectively, these findings suggest that older men might be more physically active but less socially active than women, at least in Japan. Second, associations between participation in sports groups and occupational status and marital status were observed in the present study, and those who were employed or had never been married were less likely to participate in sports groups; the findings of previous studies were inconsistent regarding the associations between physical activity and occupational status [29, 30] or marital status [18]. The association between occupation and physical activity is complex; although total physical activity includes both occupational activity and leisure–time activity [30], physical activity in sports groups is mainly leisure–time activity. Regarding marital status, spousal support for social participation might have an influence on participation in sports groups [31].

We identified, for the first time in the older Japanese population, clear socioeconomic disparities in participation in sports groups in terms of income, education, and occupation (current and previous). The greatest disparity was observed between the lowest education and highest education groups (Table 2) as well as between clerical and agricultural/forestry/fishery occupations (Table 2). These findings were similar to previous evidence from other countries [29–33], which might have important public health implications. Even in Japan, which has long been considered an egalitarian country, public health interventions to promote sports activities and social participation should consider the socioeconomic backgrounds of the target population.

Social and cultural factors had higher PRs for participation in sports groups even after adjustment for socio–demographic and health–related factors. Better social support (both receiving and providing), participation in other groups in the community, and interpersonal relationships were associated with sports group participation. Although it is unclear what mechanisms might drive these associations, social integration, including social support, contact with friends, and contact with neighbors, might encourage participation in sports groups, or it is possible that participation in sports groups encourages participation in other groups in the community by enhancing social integration [34]. Observational studies suggest that health–related characteristics, such as obesity, smoking cessation, and alcohol intake, might propagate in human social networks [35]. Thus, because social networks affect (both positively and negatively) various human behaviors, social integration might positively affect participation in sports groups. In addition, a recent review indicated that interventions using opinion leaders are effective to accelerate behavior change within a social network [36]. Thus, encouraging participation in community groups through these types of networks might promote participation in sports groups.

Regarding perceived neighborhood environments, the accessibility of facilities, access to shops, and parks and sidewalks were positively associated with participation in sports groups, which is consistent with previous studies [18, 37]. From a public health perspective, even small PRs for these environmental factors imply that changes to the neighborhood environment and related perceptions might have a significant effect on participation in sports groups.

### Study strengths and limitations

The strengths of this study include the large sample and inclusion of a wide range of variables. However, our study has several limitations. First and foremost, important factors that have been associated with physical activity were lacking, such as an individual willingness to participate in sports activities [38, 39] and previous sports participation [18,40]. Moreover, although we evaluated many variables based on a previous review of physical activity determinants [17–20], other factors might be specifically related to participation in sports groups. Second, we could not identify the types of sports groups. Third, environmental factors should ideally be evaluated and modeled as neighborhood level variables, but they were included as individual level variables in our analysis. Fourth, since most of measures, except for the population density, were self–reported, it is possible that measurement error occurred. Finally, the cross–sectional design limits any consideration of causal relationships.

### Conclusions and Implications

Future longitudinal studies or intervention studies using validated and objective measurements, such as a geographic information system for environmental evaluation (i.e., number of sports facilities) are needed to prove the causal association. Nevertheless, our findings indicate that public health interventions promoting participation in sports groups should carefully consider the socioeconomic status and social relationships of the individual as well as the neighborhood environment. In particular, participants in hobby or senior citizen clubs had higher PRs for participation in sports groups than those who did not participate in these clubs. Therefore, creating a variety of groups in the local community in which anyone can participate (e.g., men, those with less education, and agriculture/forestry/fishery workers) and encouraging participation in these groups through social networks might be effective for promoting participation in sports groups.
